# Endocytoscopic Classification Is Associated with Mucosal miR-192-5p Downregulation and CXCL2 Expression in Ulcerative Colitis: An Exploratory Study

**DOI:** 10.3390/diagnostics16152449

**Published:** 2026-08-03

**Authors:** Shungo Kanetsuki, Hiroki Kurumi, Tsutomu Kanda, Natsuki Hayashi, Takeshi Hashimoto, Ryohei Ogihara, Yu Kamitani, Yuichiro Ikebuchi, Koichiro Kawaguchi, Kazuo Yashima, Hajime Isomoto

**Affiliations:** 1 Division of Gastroenterology and Nephrology, Faculty of Medicine, Tottori University, Yonago 683-8504, Japan; shungo.kanetsuki@gmail.com (S.K.); tsutomu-k@tottori-u.ac.jp (T.K.); takeshi_hashimoto_19911022@yahoo.co.jp (T.H.); ryoheiogihara@gmail.com (R.O.); yukamitani@aol.com (Y.K.); ikebu@tottori-u.ac.jp (Y.I.); koichiro@tottori-u.ac.jp (K.K.); yashima@tottori-u.ac.jp (K.Y.); isomoto@tottori-u.ac.jp (H.I.); 2 Division of Adaptation Physiology, School of Medicine, Faculty of Medicine, Tottori University, Yonago 683-8504, Japan; hayashi-na@ncchd.go.jp

**Keywords:** endocytoscopic score, miRNA, ulcerative colitis, inflammatory bowel disease

## Abstract

**Background/Objectives**: Ultra-high-magnification endoscopy (Endocyto) visualizes microscopic mucosal structures and has been proposed as a tool for assessing mucosal inflammation. We previously developed an endocytoscopic classification system (EC-A to EC-D) for grading the severity of inflammation. In this study, we investigated the relationship between endocytoscopic classification and microRNA (miRNA) expression in 36 patients with ulcerative colitis (UC). **Methods**: This cross-sectional study was conducted in two phases at two institutions. In the first phase, microarray analysis was performed on biopsy samples from patients with UC and healthy controls enrolled at Nagasaki University Hospital. In the second phase, 36 patients with UC who underwent total colonoscopy via Endocyto at Tottori University Hospital were included, and selected miRNAs were analyzed via quantitative polymerase chain reaction. **Results**: Differentially expressed miRNAs were identified, and four (miR-141-5p, miR-192-5p, miR-194-5p, and miR-215-5p) were downregulated in inflammatory areas. Notably, miR-192-5p expression was markedly lower in the EC-B and EC-C+D groups than in the EC-A group. Furthermore, expression of CXC motif ligand 2 (CXCL2), a pro-inflammatory chemokine, was upregulated in inflammatory tissues and negatively correlated with miR-192-5p expression. **Conclusions**: These results indicate that reduced miR-192-5p expression is inversely associated with CXCL2 expression and that, among the miRNAs examined, miR-192-5p downregulation most consistently corresponded to the endocytoscopic classification. Our findings suggest that Endocyto may serve as a real-time, in vivo adjunct for a histology-like assessment of mucosal inflammation; its clinical utility requires prospective validation.

## 1. Introduction

Ulcerative colitis (UC) is a chronic idiopathic inflammatory disease of the gastrointestinal tract with an incompletely understood etiology [1,2]. UC is considered multifactorial, influenced by environmental factors, genetic predisposition, immune dysregulation, and disturbances in the gut microbiome. These factors lead to inappropriate immune activation in the lamina propria and disruption of mucosal homeostasis [2,3,4].

Genome-wide association studies have identified numerous loci associated with inflammatory bowel disease (IBD), highlighting its genetic basis. Beyond genetics, epigenetic modifications, including DNA methylation, histone modifications, long intergenic non-coding RNAs, and microRNAs (miRNAs), have emerged as key regulators of IBD pathogenesis [3,5]. miRNAs are short non-coding RNA molecules, approximately 22 nucleotides in length, that play a critical role in repressing gene expression. They bind to the 3′ untranslated region of target mRNAs, reducing mRNA stability or inhibiting translation, thereby controlling protein expression. Through this mechanism, miRNAs are central to processes such as cell differentiation, proliferation, apoptosis, and development [5]. Several miRNAs have been implicated in UC, particularly in regulating mucosal inflammation, epithelial cell turnover, and intestinal barrier function [3,5].

Ultra-high-magnification endoscopy (Endocyto OLYMPUS GIF TYPE H-290EC; Olympus Corp., Tokyo, Japan) visualizes cellular microstructures, intercellular spaces, and nuclear morphology in real time—features that are not discernible with conventional endoscopy. In IBD, this technique has shown promise in detecting subtle mucosal changes, such as goblet cell depletion, cellular distortion, and inflammation [6,7]. We previously developed the Endocyto classification (EC classification) system to grade the severity of inflammation using this imaging method and found that it correlates with histopathological findings [6,7].

Mucosal inflammation in UC is driven by various pro-inflammatory cytokines and chemokines. For example, CXC motif ligand 2 (CXCL2), a member of the CXC chemokine family, acts as a potent neutrophil chemoattractant and contributes to inflammation, wound healing, angiogenesis, and tumor progression [8]. Increased expression of CXCL2 has been reported in patients with IBD, and its expression is regulated by pro-inflammatory signaling pathways such as those mediated by tumor necrosis factor (TNF) and interleukin-17 [9,10,11,12]. CXCL2 expression is linked to miRNA regulation; for example, reduced miR-192-5p expression during active UC is associated with increased CXCL2 levels [13].

Despite these advances, the relationship between endocytoscopic findings and molecular inflammatory signatures, particularly miRNA expression, remains poorly understood. Although endocytoscopic findings correlate with histopathological inflammation, whether these ultra-high-magnification endoscopic features reflect underlying molecular alterations in the colonic mucosa remains unclear [6,7]. In particular, no studies have directly evaluated the association between endocytoscopic findings and miRNA expression profiles in patients with UC.

Clarifying whether endocytoscopic features correspond to molecular alterations in the mucosa has potential clinical relevance. Conventional endoscopic indices such as the Mayo Endoscopic Sub-score (MES) capture macroscopic mucosal appearance but may not detect residual subclinical inflammation that persists despite endoscopic remission and is associated with relapse [14]. Investigating the relationship between endoscopic scoring, including EC classification, and molecular changes in the colonic mucosa is therefore of clinical importance.

Therefore, we aimed to investigate the relationship between Endocyto findings and miRNA expression in the colonic mucosal tissue of patients with UC, as well as to determine whether endoscopic findings reflect underlying molecular changes associated with disease activity.

## 2. Materials and Methods

### 2.1. Study Design and Analytical Workflow

This was a cross-sectional observational study conducted in two phases at two institutions. In the first phase, microarray analysis of biopsy samples from patients with UC enrolled at Nagasaki University Hospital was conducted to identify candidate miRNAs potentially involved in UC pathogenesis. Based on the array results, selected miRNAs were further analyzed in the second phase using biopsy samples from a larger cohort at Tottori University Hospital.

### 2.2. Diagnostic Criteria, Eligibility, and Operational Definitions

A diagnosis of UC was established according to the diagnostic criteria of the Research Group for Intractable Inflammatory Bowel Disease (supported by the Ministry of Health, Labour and Welfare of Japan), which are based on standard clinical, endoscopic, histological, and radiological findings after the exclusion of infectious and other forms of colitis [15]. Eligible participants were patients aged ≥18 years with an established diagnosis of UC who were receiving medical treatment and underwent total colonoscopy with Endocyto. In accordance with the study protocol, the only exclusion criterion was any patient judged inappropriate for participation by the attending physician. Patients in clinical or endoscopic remission were not excluded and were sampled as described below.

Inflammatory areas (IM) and non-inflammatory areas (NIM) were defined operationally using the MES: an IM was a mucosal region with an MES ≥ 1, and a NIM was a region with an MES of 0. Paired IM and NIM biopsies were obtained only from patients with an MES ≥ 1, in whom both region types could be identified within the same colon. In patients with a global MES of 0, in whom IM and NIM could not be distinguished, two samples were obtained from NIM or from sites previously identified as IM and NIM on prior endoscopy. At each sampled area, three biopsies were obtained (one for histopathology and two for RNA analysis). Biopsies were obtained predominantly from the rectum (24 of 36 patients, 66.7%), with the remainder from the descending colon (5, 13.9%), sigmoid colon (4, 11.1%), and the transverse colon, ascending colon, and ileocecum (1 each). EC classification and MES grading were performed concurrently during the same endoscopic examination by the same endoscopist and were therefore not blinded to each other, whereas histological scoring (Matts grade and Geboes score) was performed independently by a pathologist blinded to the endoscopic findings (EC classification and MES). The four miRNAs selected for validation (miR-141-5p, miR-192-5p, miR-194-5p, and miR-215-5p) were among the most consistently and markedly downregulated in both the UC-versus-control and the inflammatory-versus-non-inflammatory comparisons in the discovery microarray. They were also selected based on biological plausibility in intestinal epithelial regulation, with miR-192-5p previously implicated in CXCL2 regulation in active UC [13].

### 2.3. Ethical Considerations

Written informed consent was obtained from all participants. This study was approved by the Nagasaki University Ethics Committee (approval number: 09062624-4; approval date: 3 March 2014) and the Tottori University Ethics Committee (approval number: 1512A094; approval date: 28 December 2015) and was performed in accordance with the ethical guidelines of the Declaration of Helsinki.

### 2.4. Patients and Materials at Nagasaki University Hospital

**Figure 1:**
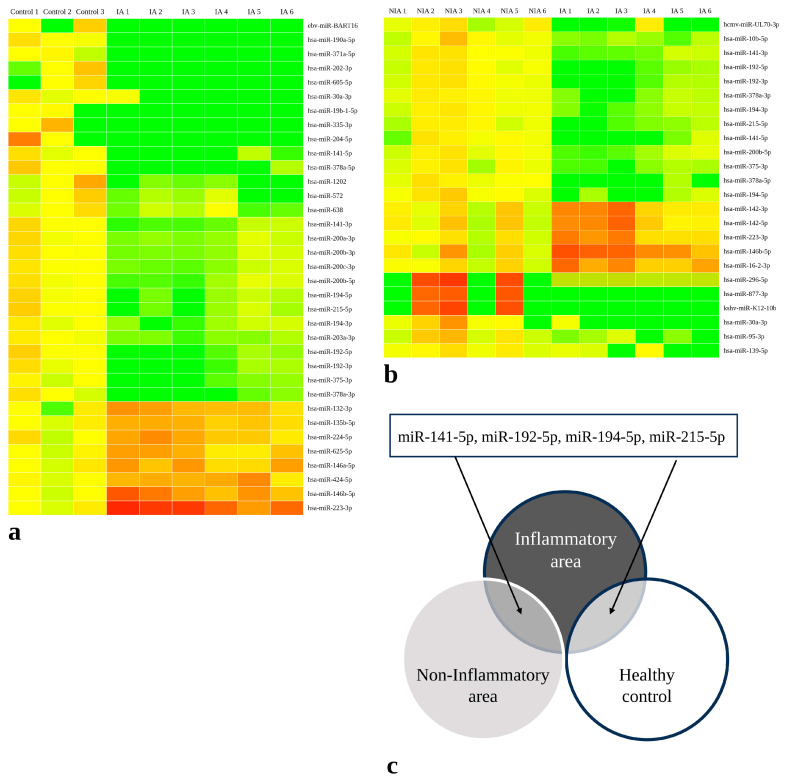
Expression profiles of candidate microRNAs in ulcerative colitis patients. (**a**) Regulation of miRNAs in the inflammatory areas of patients with ulcerative colitis when compared with the normal cohort. Expression levels were lower in the inflammatory mucosa (IM; *n* = 6) than in healthy controls (*n* = 3), as observed using unpaired *t*-test: miR-141-5p (*p* = 0.004), miR-192-5p (*p* = 0.001), miR-194-5p (*p* = 0.0064), and miR-215-5p (*p* = 0.006). (**b**) Regulation of microRNAs in the inflammatory (IM) and non-inflammatory (NIM) areas of patients with ulcerative colitis. All four miRNAs showed reduced expression in IM when compared with NIM using the Wilcoxon signed-rank test: miR-141-5p (*p* < 0.001), miR-192-5p (*p* < 0.001), miR-194-5p (*p* = 0.001), and miR-215-5p (*p* = 0.001). (**c**) Markedly downregulated miRNAs in the inflammatory areas of patients with ulcerative colitis. In the heat maps (**a**,**b**), green indicates lower and red indicates higher relative expression.

We included six patients diagnosed with UC and three healthy controls at Nagasaki University Hospital between March 2010 and September 2011. The healthy controls exhibited no gastrointestinal symptoms and underwent colonoscopy as a part of routine health screening. Biopsy samples from the three healthy controls (*n* = 3) were used for comparison with UC samples in the microarray analysis (Figure 1). Endoscopic activity was assessed in each patient with UC using the MES. As UC typically presents with rectal inflammation and proximal extension, both IM and NIM were scored using the MES, and biopsies were taken from each area. One biopsy was used for histopathology, and two for RNA analysis. Samples for histology were fixed in formalin and stained with hematoxylin and eosin (H&E). Samples for RNA analysis were immediately placed into 1 mL of RNAlater^®^ reagent (AM7021, Ambion; Thermo Fisher Scientific, Inc., Waltham, MA, USA) and stored at −80 °C until RNA isolation.

### 2.5. RNA Extraction (Discovery Cohort)

Total RNA, including small RNA, was extracted from biopsy tissues using the mirVana miRNA isolation kit (AM1560, Thermo Fisher Scientific, Waltham, MA, USA), quantified using a Nanodrop-1000 spectrophotometer (Nanodrop Technologies, Wilmington, DE, USA), purified using the miRNeasy mini kit (217004, Qiagen Co., Ltd., Tokyo, Japan), and quantified via ultraviolet absorption measurement using an Agilent 2100 Bioanalyzer (Agilent Technologies, Santa Clara, CA, USA).

### 2.6. MiRNA Array Hybridization and Analysis

Each RNA sample was analyzed comprehensively for miRNA expression patterns using microarray Rel. 16.0 (Agilent Technologies, Santa Clara, CA, USA). Differences in miRNA expression were considered significant if the fold change in expression values was >2.0 and *p* < 0.05. Multiple comparisons across the large number of miRNAs screened were addressed using the Benjamini–Hochberg false discovery rate (FDR) procedure, with an FDR-adjusted *p* value < 0.05 considered significant. The complete microarray dataset is publicly available in ArrayExpress (accession E-MTAB-16133).

### 2.7. Patients and Materials at Tottori University Hospital

We included 36 patients diagnosed with UC at the Tottori University Hospital, Tottori, Japan, who underwent total colonoscopy via Endocyto between May 2021 and February 2023. Endoscopic activity was assessed in each patient using the MES. IM and NIM were identified based on the MES scores. For paired comparisons between IM and NIM, only patients with an MES ≥ 1 were included because paired IM and NIM sampling was not feasible in patients with an MES of 0. For patients with an MES of 0, where a distinction between IM and NIM could not be discerned, tissue samples were obtained from two NIM or from previously identified IM and NIM sites, if these areas were documented in prior endoscopy. One biopsy from each area was used for histopathology and two for RNA analysis. Samples for histology were fixed in formalin and stained with H&E. RNA samples were preserved in 1 mL of RNAprotect^®^ Tissue Reagent (76106, QIAGEN) and incubated at 4 °C for 24 h. The excess reagent was removed, and samples were stored at −80 °C until RNA isolation.

### 2.8. RNA Extraction (Validation Cohort)

Total RNA was extracted from the biopsy tissues using the miRNeasy Mini Kit (217004; Qiagen Co., Ltd., Tokyo, Japan). RNA was quantified using a BioSpec-Nano spectrophotometer (206-26300-41; Shimadzu Co., Ltd., Kyoto, Japan). The extracted RNA samples were stored at −80 °C until further use.

### 2.9. MiRNA Expression Analysis Using Real-Time qPCR

Complementary DNA (cDNA) was prepared from total RNA using the miRCURY LNA RT Kit (339340; QIAGEN Co., Ltd., Tokyo, Japan). The reverse transcription (RT) reaction was performed using a reaction mixture consisting of 10 ng total RNA, 1 × reaction buffer, 1 × RT enzyme mix, and 0.5 μL RNA spike-in template. Nuclease-free water was added to a final volume of 10 μL. The reaction mixture was incubated at 42 °C for 60 min, and then at 95 °C for 5 min. Quantitative polymerase chain reaction (qPCR) was performed using a reaction mixture with a total volume of 10 μL, containing 4 μL of RT products diluted 80-fold, 5 μL of miRCURY LNA SYBR Green PCR Master Mix (339345, Qiagen Co., Ltd., Tokyo, Japan), and 1 μL of each primer. The primer sets used were from the miRCURY LNA™ miRNA PCR Assay (339306, QIAGEN Co., Ltd., Tokyo, Japan): hsa-miR-192-5p (GeneGlobe ID: YP00204099), hsa-miR-194-5p (YP00204080), hsa-miR-141-5p (YP00206080), hsa-miR-215-5p (00204598), and U6 snRNA (V2) (YP02119464). Reactions were performed using a Mic Real-Time PCR Cycler (MIC-2; Bio Molecular Systems Pty., Ltd., Tokyo, Japan). Thermal cycling was initiated at 95 °C for 10 min, followed by 45 cycles of 95 °C for 10 s and 60 °C for 60 s. The threshold cycle (Ct) was recorded for miRNA amplification using micPCR Software version 2.10.3 (Bio Molecular Systems Pty., Ltd.), and U6 was used as an endogenous control for data normalization. Relative expression was calculated using the following formula: 2 − ∆∆Ct = 2 − (∆Ct, target − ∆Ct, endogenous control).

### 2.10. mRNA Expression Analysis Using Real-Time qPCR

cDNA was prepared from total RNA using a High-Capacity cDNA Reverse Transcription Kit (4374966; Thermo Fisher Scientific, Inc., Waltham, MA, USA). Each RT reaction was performed in aliquots containing 1000 ng total RNA, 1 × RT buffer, 4 mM deoxynucleotide triphosphate mix, 1 × RT random primer, 50 units MultiScribe reverse transcriptase, and 20 units RNase inhibitor; nuclease-free water was added up to 20 μL. The reaction mixture was incubated at 25 °C for 10 min, followed by 37 °C for 120 min and 85 °C for 5 min. qPCR was performed using a total reaction volume of 20 μL containing 1 μL of 10-fold diluted RT product, 4 μL of LightCycler^®^ FastStart DNA Master PLUS SYBR Green I (03515885001, Roche Diagnostics Co., Ltd., Tokyo, Japan), 0.5 μM of each primer, and 14.6 μL of nuclease-free water. The primer sequences for qPCR were as follows: CXCL2 forward, 5′-CTCAAGAATGGGCAGAAAGC-3′ and reverse, 5′-CTTCAGGAACAGCCACCAAT-3′; and β-actin forward, 5′-CATGTACGTTGCTATCCAGGC-3′ and reverse, 5′-CTCCTTAATGTCACGCACGAT-3′. Reactions were performed using the Mic Real-Time PCR Cycler (MIC-2; Bio Molecular Systems Pty., Ltd.). Thermal cycling was initiated at 95 °C for 10 min, followed by 45 cycles of 95 °C for 10 s, 60 °C for 10 s, and 72 °C for 10 s. The Ct was recorded for mRNA amplification using micPCR software version 2.10.3 (Bio Molecular Systems Pty., Ltd.), and β-actin was used as an endogenous control for data normalization. Relative expression was calculated using the following formula: 2 − ΔΔCt = 2 − (ΔCt, target − ΔCt, endogenous control).

### 2.11. Patient Characteristics

The clinical data collected included age, sex, disease duration, clinical status, location of disease, medication, Sutherland Disease Activity Index (DAI), disease-type classification, remission, laboratory data, MES, EC classification, location of biopsy tissues, Matts histopathological grade, and Geboes histopathological score.

Disease activity and remission were defined as follows: clinical remission was defined as a Sutherland DAI ≤ 2 with no bloody stools [16,17]; endoscopic remission was an MES of 0, in line with the STRIDE-II treatment target for endoscopic healing [16]; and histological remission as a Geboes score < 3.1 (absence of neutrophils in the epithelium). The Matts histopathological grade was also recorded [18,19,20].

### 2.12. Statistical Analysis

All statistical analyses were performed using StatFlex, version 7 (Artec Co., Ltd., Osaka, Japan). Data are presented as medians with interquartile ranges, including age in both cohorts. Apart from the unpaired *t*-test used for candidate miRNA selection in the discovery microarray phase, nonparametric tests were used because the data were not normally distributed. Comparisons between paired samples and two independent groups were performed using the Wilcoxon signed-rank and Mann–Whitney U tests, respectively. For comparisons among more than two groups, the Kruskal–Wallis test followed by Dunn’s multiple comparison test was applied. Correlations were assessed using Spearman’s rank correlation coefficient. All tests were two-tailed, and *p* values < 0.05 were considered statistically significant. Given the exploratory nature of the study, the sample size was determined by feasibility, and the findings should be considered hypothesis-generating.

## 3. Results

### 3.1. MicroRNA Screening in the Discovery Cohort

The miRNA expression levels were compared between IM and NIM in patients with UC treated at Nagasaki University Hospital, and between patients with UC and healthy controls. In the initial microarray screening, miRNA expression profiles were compared between UC samples (*n* = 6) and healthy controls (*n* = 3) (Figure 1). Differentially expressed miRNAs were identified using a fold change > 2.0 and an uncorrected *p* < 0.05; however, no miRNA remained statistically significant after Benjamini–Hochberg FDR correction. The four candidate miRNAs (miR-141-5p, miR-192-5p, miR-194-5p, and miR-215-5p), which had the lowest uncorrected *p* values (*p* = 0.002–0.004) and biological relevance to UC, were among the most highly ranked in the inflammatory-versus-non-inflammatory comparison and were therefore selected for validation by qPCR in an independent cohort. All patients in the UC group had either recurrent or active UC (Table 1). Levels of miR-141-5p, miR-192-5p, miR-194-5p, and miR-215-5p were markedly downregulated in IM when compared with the NIM. Similarly, the UC group exhibited lower expression of these miRNAs than the healthy control group (Figure 1).

### 3.2. Characteristics of the Validation Cohort

Biopsy samples were collected from 36 patients at Tottori University Hospital. Paired comparisons between IM and NIM were performed in a subset of 24 patients with an MES ≥ 1. Samples were collected from both IM and NIM to examine the relationship between the selected miRNAs and the pro-inflammatory chemokine CXCL2. The 36 patients were categorized into EC-A (*n* = 11), EC-B (*n* = 18), EC-C (*n* = 5), and EC-D (*n* = 2) groups. An MES ≤ 1 was present in 69% of patients, and endoscopic remission (an MES of 0) in 33.3% (12 of 36); clinical remission was present in 67% (Table 2). When EC-C and EC-D were analyzed as separate categories, the direction of change in miR-192-5p and CXCL2 was consistent with that of the combined EC-C+D analysis, but the individual subgroups were underpowered. Compared with EC-A, the difference for EC-C was not statistically significant, whereas EC-D showed a significant difference; however, this finding was based on only two patients. In this predominantly mild cohort, few patients showed the EC-D endoscopic findings. EC-C and EC-D were therefore combined into a single EC-C+D category for the analyses.

### 3.3. MicroRNA Expression by Inflammation, MES, and EC Classification

**Figure 2:**
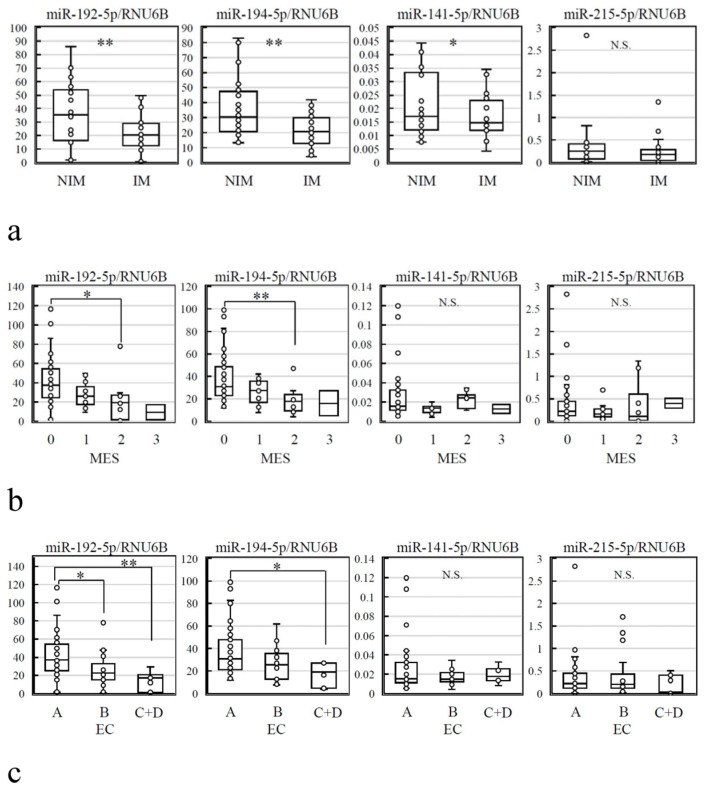
Expression levels of miR-141-5p, miR-192-5p, miR-194-5p, and miR-215-5p. (**a**) Comparison between expression in inflammatory (IM) and non-inflammatory (NIM) areas (*n* = 24 pairs). miR-192-5p, miR-194-5p, and miR-141-5p were lower in the IM than in NIM area (Wilcoxon signed-rank test; miR-192-5p *p* < 0.001, miR-194-5p *p* < 0.001, miR-141-5p *p* = 0.02), while miR-215-5p showed no difference (*p* = 0.07). (**b**) Expression levels according to the Mayo Endoscopic Sub-score (MES). miR-192-5p and miR-194-5p were lower in MES 2 than in MES 0 (Dunn’s multiple comparison test; miR-192-5p *p* = 0.02, miR-194-5p *p* = 0.007). No differences were observed among the remaining MES comparisons for miR-141-5p (*p* = 0.37) or miR-215-5p (*p* = 0.80). (**c**) Expression levels according to endocytoscopic classification (EC). miR-192-5p was lower in EC-B and EC-C+D when compared with EC-A (Dunn’s multiple comparison test; EC-B vs. EC-A: *p* = 0.04, EC-C+D vs. EC-A: *p* = 0.003). miR-194-5p was reduced in EC-C+D versus EC-A (*p* = 0.011). No differences were observed among EC classifications for miR-141-5p (*p* = 1.00) or miR-215-5p (*p* = 0.33). Boxes represent the median and interquartile range, whiskers represent the range, and circles represent individual data points; * *p* < 0.05, ** *p* < 0.01.

Levels of miR-192-5p, miR-194-5p, and miR-141-5p were lower in IM than in NIM (Figure 2a). Among the MES groups, the expression levels of miR-192-5p and miR-194-5p were lower in the MES 2 group than in the MES 0 group (Figure 2b). No significant differences were observed across MES groups for miR-141-5p and miR-215-5p. Analysis of miRNA expression based on EC classifications showed that only miR-192-5p expression was significantly lower in the EC-B and EC-C+D groups than in the EC-A group (Figure 2c).

### 3.4. CXCL2 Expression and Its Correlation with miR-192-5p

**Figure 3:**
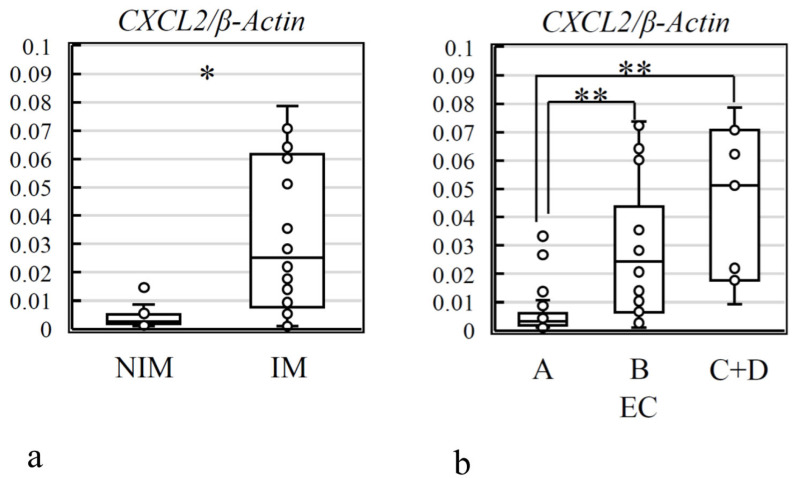
Expression levels of CXC chemokine ligand 2 (CXCL2) in inflammatory and non-inflammatory areas and across EC classifications. (**a**) Comparison between inflammatory (IM) and non-inflammatory (NIM) areas (*n* = 24 pairs). CXCL2 expression was higher in the IM than in NIM area (Wilcoxon signed-rank test; *p* < 0.001). (**b**) Expression levels according to endocytoscopic classification (EC). CXCL2 expression was higher in EC-B and EC-C+D when compared with EC-A (Dunn’s multiple comparison test; EC-B vs. EC-A: *p* < 0.001, EC-C+D vs. EC-A: *p* < 0.001). Boxes represent the median and interquartile range, whiskers represent the range, and circles represent individual data points; * *p* < 0.05, ** *p* < 0.01.

**Figure 4:**
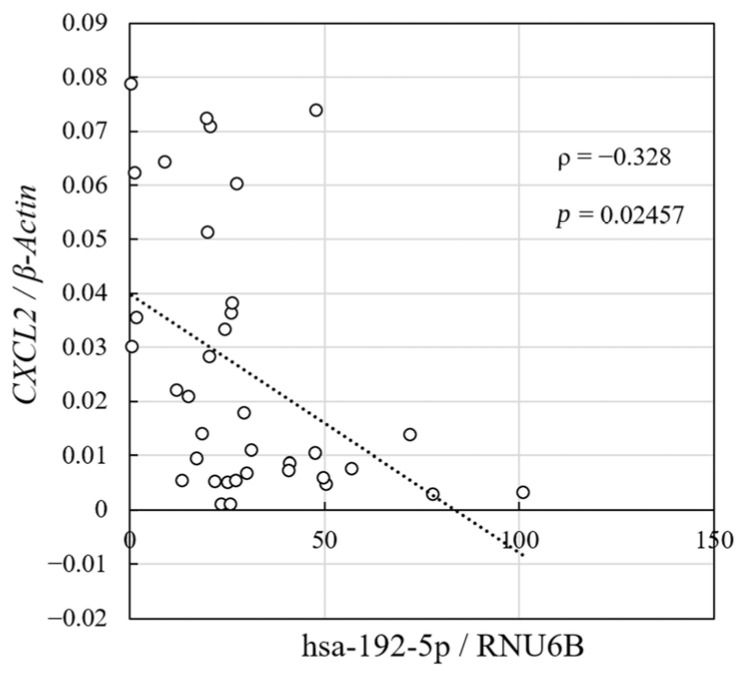
Correlation analysis between miR-192-5p expression and CXCL2 expression. A negative correlation was observed between miR-192-5p and CXCL2 expression in inflammatory mucosa (Spearman’s rank correlation; ρ = −0.328, *p* = 0.02).

Expression levels of the pro-inflammatory chemokine CXCL2 were higher in IM than in NIM. CXCL2 expression was also increased in the EC-B and EC-C+D groups compared with that in the EC-A group (Figure 3). A negative correlation was observed between miR-192-5p expression and CXCL2 expression (Figure 4). The inverse correlation was statistically significant (Spearman’s ρ = −0.328, *p* = 0.02).

### 3.5. Correlation of miR-192-5p and CXCL2 with Histological Grade

**Figure 5:**
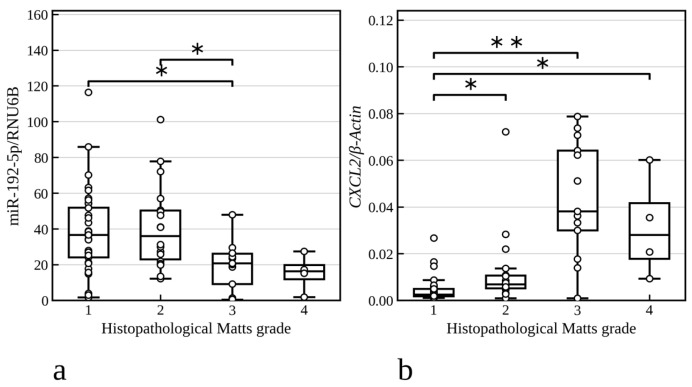
Expression of miR-192-5p and CXCL2 according to the Matts histopathological grade. (**a**) miR-192-5p/RNU6B and (**b**) CXCL2/β-actin relative expression across Matts grades 1–4 (*n* = 72 biopsy samples). Boxes represent the median and interquartile range, whiskers represent the range, and circles represent individual samples. miR-192-5p decreased and CXCL2 increased with increasing histological activity (Spearman ρ = −0.331, *p* = 0.005 and ρ = 0.677, *p* < 0.001, respectively). * *p* < 0.05, ** *p* < 0.01 (Dunn’s multiple comparison test).

To relate the molecular findings to histological and endoscopic activity, miR-192-5p and CXCL2 expressions were examined across the Matts histopathological grade and the EC classification (*n* = 72 biopsy samples). miR-192-5p expression was progressively lower, and CXCL2 expression progressively higher, at higher Matts grades (Figure 5a,b; Dunn’s multiple comparison test). Consistent with these grade-wise differences, Spearman rank correlation showed that miR-192-5p was inversely, and CXCL2 positively, correlated with the Matts grade (ρ = −0.331, *p* = 0.005 and ρ = 0.677, *p* < 0.001, respectively) and with the EC classification (ρ = −0.440 and ρ = 0.638; both *p* < 0.001).

## 4. Discussion

In this study, we investigated the relationship between Endocyto findings and miRNA expression in the colonic mucosal tissue of patients with UC, and determined whether endoscopic findings reflect underlying molecular changes associated with disease activity. We previously proposed the EC classification using ultra-high-magnification endoscopy and reported that it correlated with histopathological findings in patients with UC [6]. Accordingly, in the current study, we used the EC classification, which comprises four categories: EC-A, regular arrangement of round-to-oval pits; EC-B, irregular arrangement with or without enlarged spaces between otherwise regular pits; EC-C, deformed pits with distorted crypt lumens that are irregularly arranged but remain intact; and EC-D, disrupted or absent pits [6]. As previously demonstrated, the EC classification is associated with clinical relapse rates. Furthermore, achieving EC-A has been linked to a reduced relapse rate during long-term follow-up [7].

In a small cohort study conducted at the Nagasaki University Hospital, four candidate miRNAs (miR-141-5p, miR-192-5p, miR-194-5p, and miR-215-5p) were identified as potential targets. Subsequently, the expression of these four miRNAs was analyzed in a larger cohort at Tottori University Hospital. The MES is a widely used index for evaluating mucosal inflammation in patients with UC. Although some miRNAs differed between MES groups, none of the four miRNAs demonstrated a consistent trend across all MES categories. In contrast, miR-192-5p expression was lower in the inflammatory groups (EC-B and EC-C+D) than in the non-inflammatory EC-A group (Figure 2). Among the four candidate miRNAs, only miR-192-5p was consistently associated with EC classification, whereas miR-141-5p and miR-215-5p showed no significant association and miR-194-5p showed only a partial association. The apparently clearer discrimination of molecular differences by EC classification than by the MES should be interpreted cautiously, because it may be influenced by the characteristics of the present cohort, which contained relatively few patients with high MES scores and a high proportion of patients in clinical remission, thereby limiting the detection of MES-related differences. We therefore do not claim that EC classification is superior to validated endoscopic indices; rather, the two may provide complementary information.

The downregulation of miR-192-5p and upregulation of the pro-inflammatory chemokine CXCL2 were observed in both IM versus NIM and among EC groups. Additionally, a negative correlation was observed between miR-192-5p expression and CXCL2 expression in the colonic mucosa of patients with UC. This inverse correlation is consistent with a previous report of reduced miR-192-5p expression accompanying increased CXCL2 expression in active UC [13]; the correlation was modest, and the causal relationship between miR-192-5p and CXCL2 remains to be determined in future studies. These results suggest that endocytoscopic findings align with the expression patterns of inflammation-related molecules such as miR-192-5p and CXCL2, showing that EC classification corresponds with histology-like features and selected molecular markers of mucosal inflammation. Consistent with these observations, miR-192-5p expression was lower and CXCL2 expression higher at higher Matts histopathological grades (Figure 5), and the two markers were, respectively, inversely and positively correlated with the grade. However, these associations differed in strength, being clearer for CXCL2 than for miR-192-5p, and not all pairwise comparisons between grades reached statistical significance, partly reflecting the small number of patients in the higher grades. These findings therefore suggest a trend toward an association between the molecular changes and histological activity rather than a consistent parallel, and no causal relationship can be inferred.

The precise mechanism by which miR-192-5p regulates CXCL2 remains unclear; however, studies have shown that miR-192-5p is regulated by transforming growth factor-beta (TGF-β), p53, and SMAD family member 3 (SMAD3) signaling pathways [21,22,23]. miR-192-5p plays a role in intestinal autophagy and mucosal healing, and SMAD3 knockout induces intestinal inflammation in mice [24,25,26]. Although studies have implicated miR-192-5p in inflammatory pathways, the present study did not directly assess its mechanistic role. Therefore, whether reduced miR-192-5p expression contributes to inflammatory processes in UC remains to be clarified.

This study has some limitations. First, the sample size was relatively small and the cohort was skewed toward mild disease, with 67% of patients in clinical remission. This limited the statistical power and our ability to evaluate molecular expression across the full spectrum of disease activity, particularly in moderate-to-severe cases. Second, the healthy control group comprised only three individuals, and no miRNA remained significant after FDR correction; the discovery microarray findings should therefore be considered exploratory and hypothesis-generating, and the comparison between UC and normal mucosa should be interpreted with caution. Third, the molecular analysis was restricted to selected miRNAs and a single target mRNA (CXCL2), and no functional experiments (such as miR-192-5p gain- or loss-of-function or direct target validation) were performed; the data therefore demonstrate associations only and cannot establish a causal or regulatory relationship between miR-192-5p and CXCL2 or address other epigenetic and inflammatory pathways. Fourth, because only patients who underwent Endocyto examination at two Japanese institutions were included, selection bias cannot be excluded, the findings may not be generalizable to populations with different genetic or environmental backgrounds, and they lack external validation. Fifth, the cross-sectional design cannot determine whether miR-192-5p downregulation or endocytoscopic changes precede clinical deterioration or respond to treatment, and no clinical outcomes (relapse, treatment response, treatment escalation, hospitalization, or colectomy) were assessed; the clinical relevance of the observed associations therefore remains to be established in prospective studies. Finally, EC classification and MES were assessed by the same endoscopist and were not blinded to each other, which may have introduced observer bias, whereas histological scoring was performed by a pathologist blinded to the endoscopic findings.

Despite these limitations, our findings suggest that endocytoscopic findings were associated with both histological features and the expression of selected molecular markers in UC. According to the *Selecting Therapeutic Targets in Inflammatory Bowel Disease* guidelines, endoscopic mucosal healing is a formal treatment target [16], whereas histological healing remains an aspirational goal. However, evidence suggests histological healing may better predict long-term outcomes. For example, Liang et al. reported that patients in clinical remission without histological healing had a significantly higher relapse rate (26.1% vs. 50.0%, *p* = 0.028) and shorter relapse-free survival (46.1 months vs. 31.5 months, *p* = 0.015) [14]. In the context of such evidence, modalities that correlate with histological status, including EC classification, may hold clinical relevance.

## 5. Conclusions

Our findings indicate that differences in EC classification are associated with variations in miR-192-5p and CXCL2 expression in the colonic mucosa of patients with UC. These results show that endocytoscopy corresponds with histological features and, among the miRNAs examined, specifically with miR-192-5p. Endocyto may serve as a real-time, in vivo adjunct for a histology-like assessment of mucosal inflammation in UC; however, its clinical utility requires prospective validation through further functional and longitudinal studies.

## Figures and Tables

**Table 1:** Clinical characteristics of six patients with ulcerative colitis at the time of endoscopic observation at Nagasaki University Hospital.

Characteristics	*n* = 6
Age, years, median (IQR)	35.5 (30.75–42.75)
Sex, male/female	2/4
Clinical Status	
Remission	0
Active	6
Location of disease, *n* (%)	
Left-side colitis	1 (16.7)
Proctitis	5 (83.3)
Treatment, *n* (%)	
5-ASA	5 (83.3)
5-ASA + Oral PSL + Topical PSL	1 (16.7)
Endoscopic Matts Grade, *n* (%)	
1	0
2	6 (100)
3	0
4	0

5-ASA = 5-aminosalicylic acid, PSL = prednisolone, IQR = interquartile range.

**Table 2:** Clinical characteristics of 36 patients with ulcerative colitis at the time of endoscopic observation at Tottori University Hospital.

Characteristics	*n* = 36
Age, years, median (IQR)	43.0 (34.50–55.5)
Sex, male/female	24/12
Duration of disease, yr, median (IQR)	5.58 (2.15–13.7)
DAI, median (IQR)	1 (0–3)
Remission, *n* (%)	
Remission	24 (67)
No-remission	12 (33)
Location of disease, *n* (%)	
Pancolitis	20 (55.6)
Left-side colitis	11 (30.6)
Proctitis	5 (13.9)
Treatment, *n* (%)	
5-ASA	13 (36.1)
5-ASA + Oral PSL	3 (8.3)
5-ASA + Topical PSL	2 (5.6)
5-ASA + IMM	2 (5.6)
5-ASA + IMM + Oral PSL	1 (2.8)
5-ASA + IMM + Bio	5 (13.9)
5-ASA + Topical PSL + IMM + Bio	1 (2.8)
5-ASA + Bio	3 (8.3)
Oral PSL	1 (2.8)
Topical PSL	1 (2.8)
IMM + Oral PSL	1 (2.8)
IMM + Bio	1 (2.8)
Probiotics	1 (2.8)
No treatment	1 (2.8)
Laboratory data, median (IQR)	
WBC, /µL	5500 (4700–6325)
CRP, mg/L	0.05 (0.03–0.10)
ESR, mm	4.5 (2–8)
MES, *n* (%)	
0	12 (33.3)
1	13 (36.1)
2	9 (25.0)
3	2 (5.6)
EC classification, *n* (%)	
A	11 (30.6)
B	18 (50.0)
C	5 (13.9)
D	2 (5.6)
Location of biopsy tissues, *n* (%)	
Ileocecum	1 (2.8)
Ascending colon	1 (2.8)
Transverse colon	1 (2.8)
Descending colon	5 (13.9)
Sigmoid colon	4 (11.1)
Rectum	24 (66.7)
Matts histopathological grade, *n* (%)	
1	1 (2.8)
2	18 (50.0)
3	13 (36.1)
4	4 (11.1)
Geboes histopathology score, *n* (%)	
<3.1	3 (8)
≥3.1	33 (92)

5-ASA, 5-aminosalicylic acid; Bio, biologics; CRP, C-reactive protein; DAI, disease activity index; EC, endocytoscopic; ESR, erythrocyte sedimentation rate; IMM, immunomodulator; IQR, interquartile range; MES, Mayo endoscopic sub-score; PSL, prednisolone; WBC, white blood cell.

## Data Availability

The datasets generated and/or analyzed during the current study can be made available from the corresponding author on reasonable request. The microarray dataset has been deposited in ArrayExpress (accession: E-MTAB-16133) and is publicly available. Other data are available from the corresponding author upon request.

## Abbreviations

The following abbreviations are used in this manuscript:

Endocyto	Ultra-high magnification endoscopy
UC	Ulcerative colitis
CXCL2	CXC motif ligand 2
IBD	Inflammatory bowel disease
TNF	Tumor necrosis factor
MES	Mayo Endoscopic Sub-score
H&E	Hematoxylin and eosin
cDNA	Complementary DNA
RT	Reverse transcription
qPCR	Quantitative polymerase chain reaction
DAI	Sutherland Disease Activity Index
SMAD3	SMAD family member 3
EC	Endocytoscopic classification
IM	Inflammatory area
NIM	Non-inflammatory area
miRNA	MicroRNA
FDR	False discovery rate
IQR	Interquartile range
